# Trend of invasive pneumococal disease (IPD) in a South Western, Nigerian hospital

**DOI:** 10.11604/pamj.2016.23.140.5635

**Published:** 2016-03-28

**Authors:** Motayo Babatunde Olanrewaju, Akingbade Olusola, Nwadike Victor, Shobayo Olabode, Ogiogwa Joseph, Akinduti Akiniyi, Okonko Iheanyi

**Affiliations:** 1 Medical Microbiology Unit, Pathology Department, Federal Medical Centre, Abeokuta, Nigeria; 2 Department of Microbiology, Federal University of Agriculture, Abeokuta, Nigeria; 3 Department of Vetenary Microbiology and Parasitology, Federal University of Agriculture, Abeokuta,Nigeria; 4 Department of Microbiology, University of Port-Harcort, Nigeria

**Keywords:** Immunization, streptococcus pneumonia, IPD, Abeokuta

## Abstract

The recent introduction of the Heptavalent-pneumococcal vaccine (PCV-7) by private pharmaceutical companies in Nigeria, has generated interest in invasive bacterial diseases particularly IPD. Our objective in this study is to investigate the trend and occurrence rate of IPD in Abeokuta, Nigeria. Suspected IPD cases were assessed from Jan 2010 to Dec 2010 for demographic and Microbiological characteristics. Bacterial isolations and antibiotics susceptibility testing followed standard bacteriological procedure. Overall 471 cases of probable IPD was assessed, with 21(4.5%) cases of suspected pneumonia, 109(23.1%) cases of suspected meningitis, and 341(72.4%) cases of suspected septicaemia. Confirmed IPD cases were 9 with 2 cases of meningitis, 3 cases of septicaemia and 4 cases of pneumonia. Age range distribution showed, high distribution of IPD cases among children >1 with 5(55.6%) there was a statistically significant difference in gender p< 0.05 (X2 test) with females recording a higher occurrence than males. We conclude by advocating for better detection methods against IPD meningitis cases, and continuous surveillance into the serotypes of streptococcus pneumonia as well inclusion of the PCV vaccine into our childhood immunization program.

## Introduction

Streptococcus pneumoniae or pneumococcus is a gram positive cocci bacterium responsible for a variety of infectious diseases, some of which are very invasive and life threatening such as Meningitis, Pneumonia and blood stream infections. They are responsible for over 4 million illnesses in the United States alone [1]. Reports have also shown that S. pneumoniae are responsible for over 1 million deaths of > 5 children annually [2]. The introduction of the 23-valent polysaccharide vaccine giving protective coverage to 23 serotypes to the over 90 circulating serotypes gave a relive by its protective role against Invasive Pneumococcal Disease (IPD) in most at-risk children in developed countries [3]. Reports of insufficient immunogenicity and insufficient protective coverage particularly in infants lead in 2001 to the introduction of the improved heptavalent pneumococcal-conjugate vaccine PCV-7 which is more immunogenic and offers good coverage against the commonest serotypes of s. pneumoniae, 4, 6B, 9V, 14, 18C, 19F and 23F in childhood immunization programs in most developed countries [3, 4]. Since the introduction of the PCV vaccine in childhood immunization programs, implementing countries have experienced a reduction in mortality caused by IPD and a reduced incidence rate in the number of IPD cases [4]. Owing to the limited coverage capacity of PCV-7, there have been reports of increase in IPD cases caused by non PCV-7 serotypes [5, 6]. In Nigeria there is reported evidence of relatively high IPD related disease burden, a recent study in Ibadan, revealed that 39.3% of a total of 1210 cases of probable IPD were meningitis and 33% of that figure were pneumonia [2]. The recent introduction of the PCV vaccine by Glasgow Smithkline (GSK) a multi-national pharmaceutical company in Nigeria seems to bring relief, but the high cost of procurement has seem to put it out of the reach of the most at risk population. The introduction of the PCV in Nigeria still needs government intervention as it is yet to be included in EPI (Expanded program on Immunization) program. Research into the actual disease burden and epidemiological pattern including the most prevailing serotypes in Nigeria needs to be stepped up, with the introduction of this new vaccine. Our objective has been motivated by this need, this study was conducted to retrospectively review IPD cases, and their demographic and bacteriological pattern in Abeokuta, South west, Nigeria.

## Methods

### Study site and study design

The study was carried out at the Federal Medical Centre, Abeokuta, Ogun State, Nigeria. The hospital is a growing tertiary Institution with about 400 beds and specialist wards including a functional pediatric ward and pediatric intensive care unit. The hospitals serves as a referral centre for Ogun state and neighboring Lagos State, because of the teeming population of Lagosians. The study is a review of all suspected and Laboratory confirmed IPD cases in F.M.C Abeokuta from January 2010 to December 2010. Relevant information was retrieved from laboratory records of the Medical Microbiology and Parasitology Unit of the Center. All information regarding patient's data was accorded the highest level of confidentiality in accordance to the Belmont report (Document of the U.S dept of Education Health and welfare, 1979).

### Case definitions

For this study clinical definitions were used, for a suspected or probable case of IPD, it was defined as any case of Pneumonia, Septicaemia or Meningitis, followed up by a Microbiology analysis but without a positive culture result or with a positive culture result not yielding Streptococcus pneumoniae. A confirmed IPD case was defined as a suspected case of Pneumonia, septicaemia, or Meningitis yielding a positive culture result with Streptococcus pneumoniae, from a normally sterile site such as blood or cerebrospinal fluid c.s.f [7].

### Laboratory analysis

All samples were processed at the Medical Microbiology Unit of F.M.C. Abeokuta. Samples submitted fro processing included, cerebrospinal fluid, (c.s.f), blood, pleural aspirate. For c.s.f samples, the sample was examined macroscopically for appearance, volume and presence of blood. Microscopic examination was done by wet preparation for presence of leucocytes, and direct gram stain for demonstration of bacteria. The c.s.f sample was then cultured according to standard bacteriologic procedure [8] and isolates were identified as Streptococus.p by haemolysis on blood agar (Sheep blood) and susceptibility to optocin [8]. For blood, inoculated blood cultre bottles were incubated at 370C for 4 to 7 days with 3 sub cultures as previously described [9]. All isolates were identified using standard microbiology techniques [8]. Pleural aspirate was processed following standard bacteriological technique. All isolates were tested for antibiolotic susceptibility following the Kirby-Bauer method and results were interpreted using CLSI break points [8, 10].

Statistical analysis: Data generated was, averaged and organized into tables, comparisons were made using the chi square test for parametric variables and a p-value of less 0.05 (p> 0.05) was set as significant.

## Results

**Figure 1:**
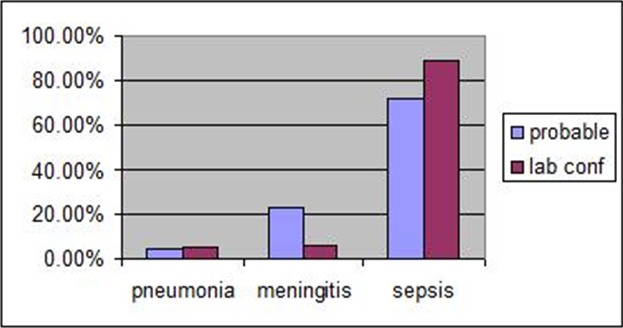
Bar gragh showing the distribution of probable and laboratory confirmed Invasive Bacteria disease cases at FMC Abeokuta

During the period under review, a total of 471 cases of probable IPD, with a total of 21(4.5%) cases of suspected pneumonia, 109(23.1%) cases of suspected meningitis, and 341(72.4%) cases of suspected septicaemia. The number of confirmed cases are shown in Figure 1 with 6(5%) cases of pneumonia 7(5.9%) of meningitis and 106(89.1%) of confirmed septicaemia. The demographic distribution containing age and gender distribution of probable IPD cases and IPD confirmed cases reveals that, they were almost twice as more females than males who presented with probable IPD cases with males recording 187(39.7%) and females 284(60.3%) age group distribution showed that less than 1 year old children consisted more than half of the total probable IPD cases with 263(55.8%) age group 1 > 15 recorded 173(36.7%) and lastly 15 and above with 35(7.5%) details of confirmed cases are shown in Table 1. Distribution of isolated microorganisms from various invasive bacterial diseases including IPD cases in relation to diagnosis is shown below in Table 2 with Streptococcus pneumonia recording 3(2.7%) isolation rate against septicaemia, 4(66.7%) of total isolation against pneumonia and 2(33.3%) of total isolation against meningitis. Staphylococcus aureus recorded 44(41.5%) of total isolations against septicemia making it the highest bacteria species isolated in the reviewed invasive bacterial diseases, no isolations of s. aureus were made against pneumonia and 2(33.3%) isolation was recorded amongst meningitis cases. Details of other bacteria isolations such as escherichai coli, klebsiela pneumomiae and other types of bacteria are shown in Table 2 below. Table 3 shows the antibiotic susceptibility pattern of the 9 isolates of s. pnuemoniae recovered in this study.

**Table 1:** Demographic distribution showing age and gender distribution of suspected/probable IPD cases and culture confirmed IPD cases

Gender	Probable cases N (%)	Confirmed cases N (%)
Male	187 (39.7)	3 (33.3)
Female	284 (60.3)	6 (66.7)
Age group		
>1	263 (55.8)	5 (55.6)
1>15	173 (36.7)	2 (22.2)
15>	35 (7.5)	2 (22.2)
Total	471 (100)	9 (100)

**Table 2:** Distribution of bacteria isolates recovered from various Invasive bacteria diseases in relation to Clinical diagnosis including IPD isolates

Organism	Clinical diagnosis		
	Septicaemia	Pneumonia	Meningitis
*Strep. Pneumonia*	3 (2.7)	4 (66.7)	2 (33.3)
*Staph. Aureus*	44 (41.5)	0 (0)	2 (33.3)
*E. coli*	29 (27.4)	0 (0)	1 (16.6)
*Kleb. Pneumonia*	15 (14.2)	2 (33.3)	0 (0)
Others	15 (14.2)	0 (0)	1 (16.8)
Total	106 (100)	6 (100)	6 (100)

**Table 3:** Antibiotic susceptibility profile of *Streptococcus pneumonia* tested against some commonly prescribed antibiotics in Abeokuta, Nigeria

Age	Diagnosis	Sample	Susceptibility	
			Susceptible	Resistant
2yrs	Sepsis	Blood	Ery, Tet, Gen	Amox, Amox/clav
6mths	Meningitis	C,S,F	Ery, Amox, Amox/clav	Tet, Gen
1wk	Sepsis	Blood	Amox, Amox/clav, Gen	Tet
1yr	Meningitis	C.S.F	Gen, Ery, Amox/clav	Tet, Amox
5yrs	Pneumonia	Throat swab	Gen, Ery, Amox/clav	Tet, Amox
11yrs	Pneumonia	Aspirate	Amox, Amox/clav, Ery	Tet, Gen
8yrs	Pneumonia	Aspirate	Gen, Ery, Amox/clav	Amox, Tet
9mths	Sepsis	Blood	Gen, Amox/clav	Ery, Tet, Amox
19yrs	Pneumonia	Aspirate	Amox/clav	Gen, Ery, Tet, Amox

Keys: Ery- Erythromycin, Tet-Tetracycline, Gen-Gentamycin, Amox-Amoxcillin, Amox/clav-Amoxicillin/clavunalate.

NB: Antibiotics tested include Amox/clav 30µg, Erythromycin (25µg), Amoxicillin (25µg), Tetracyclie (30µg), Cotrimaxole (25µg), Gentamycin (10µg) produced by (Abtek biologicals UK.)

## Discussion

Our study highlights the occurrence rate of suspected IPD cases in Abeokuta, Nigeria remains high with our current estimate reaching 471 cases through the year 2010. This is much lower than that of a recent multi-centre study done at Ibadan which reported about 171 probable cases with 21 culture confirmed cases [2]. The number of culture positive cases in our study is about a third of the number of specimens received (119 cases) judging that each case patient submitted at least one culture sample for processing. The number of culture confirmed cases in our study 9(7.6%), seems unrealistically low comp aired to some reports from advanced countries [7, 11]. The very low recovery rate of the incriminating pathogen however is not unique to our own study setting as very low recovery rates were also observer in a study done at Ibadan, within the same geo-political zone as ours [2]. Factors responsible for low rates observed in our current study include antibiotic usage prior to sample collection, poor communication and sample transportation particularly for C.S.F samples, and low sensitivity rate with the culture techniques used, for instance human blood was used instead of sheep blood in preparing the media used for isolation of pathogens. Similar constraints have been previously reported [2] in our environment; this has made it difficult to make any justifiable comparison to other studies done outside our environment. Septicaemia was the most frequently diagnosed disease with 314(72.4%) cases, followed by Meningitis 109(23.1%) of diagnosed cases Pneumonia was the least observed case, this is in agreement with the report of Falade et al., [2] which recorded a higher Meningitis case detection rate than pneumonia, this observed trend is also concomitant to other reports that gave the same trend [7, 11]. Demographic distribution shows that females recorded a higher occurrence rate than males, p < 0.05 (Chi square test), this might be strictly co-incidental, because previous reports have indicated male gender is a risk factor for severe Respiratory Syncytial virus (RSV) infection [12]. Age group distribution revealed that children of > 1yr old were the most affected with 263(55.8%) of probable cases and 5(55.6%) of culture confirmed cases, this is in agreement with the report of Shaidi et al. [3], but is not with agreement with another report that indicated a higher infection rate in the elderly above 65 years old in the Netherlands [4]. Surveillance data have shown that majority of Invasive respiratory diseases are higher at the 2 extremes of life, however we do not know exactly why a low rate was observed in our current study, it is worthy of note to mention that many of the senior citizens’ in our environment because of financial constrains and ethno-religious beliefs do not visit the hospital until when situations gets out of hand.

Perhaps a more robust cohort study is needed to investigate this finding. The occurrence rate of recovered bacteria isolates in our current study shows that Septicaemia had the highest recovery rate, with staphylococcus aureus being the commonest pathogen, this could be as a result of sample contamination, although in the current study proper care was taken to reduce the incidence of contamination among our samples. Pneumonia cases however had streptococcus pneumonia being the most frequently recovered isolate. A major limitation which would have had added significant epidemiological value to this work was our inability to demonstrate the existing serotypes of S. pneumonia strains. Our laboratory was not able to procure necessary serotyping reagents during the period under study, this is however regretted. Previous report has however shown the existance of serotypes 5, 19F and 4 circulating within our geographical region [2]. Antibiotic susceptibility pattern revealed that of the 9 isolates of s. pneumonia recovered, majority were resistant to at least 2 classes of antibiotics Table 3. This is a worrisome trend as there have been several reports of emerging beta-lactamase resistance in our study environment [13–15]. There has been evidence of the presence of plasmid transmissible multiresistant resistant bacteria isolates within hospital subjects, this puts pressure on colonsing otherwise susceptible bacteria and could be responsible for the currently observed trend [13, 16].

## Conclusion

The current study has revealed relatively high rates of Invasive bacterial diseases, including Meningitis and Pneumonia in Abeokuta, southwest, Nigeria. Our inability to show the serotype distribution of isolated strains of S. pneumonia is however regretted. Our study has indicated an emerging multi-drug resistance MDR trend among s. pneumonia recovered in our study environment, this again calls for responsible antibiotic stewardship even among previously susceptible pathogens. Better s. pneumoniae detection methods particularly for Meningitis cases such as the Immunochormatographic antigen detection tests are advocated for routine use in other to increase detection of otherwise culture negative cases. We also call for more efforts from concerned authorities for increased surveillance against IPD in our environment and government's full inclusion of PCV-7 into the routine childhood immunisation programme.

### What is known about this topic

- Invasive streptococcal diseases are an established disease condition in Nigeria, with a significant mortality rate.
- Pneumococcal vaccine PCV 13 by GSK is already available for use in Nigeria.
- There are diagnostic challenges regarding rapid detection of IPD particularly in cases of childhood meningitis.

### What this study adds

- The current study reveals the presence of drug resistance streptococcus pnuemoniae in Abeokuta, Nigeria.
- Our study highlights the importance of introduction of rapid diagnostic test to achieve faster detection and better clinical outcome.
